# Medical Opinion and Movement

**Published:** 1907-12-28

**Authors:** 


### Medical Opinion and Movement.

In a recent issue we called attention to the splendid example that has been set by the authorities at Huddersfield in their organised and successful efforts to reduce the infant mortality, and we expressed the hope that this example might be followed in other communities. Dr. A. E. Harris, Medical Officer of Health for Islington, has drawn the attention of the Borough Council to the matter by an excellent report, in which he discloses a deplorable death-rate among the infant population of the district, places his finger on the causes, and indicates the lines on which remedial measures should be adopted. An examination of the mortality figures shows that the first week is by far the most critical period. In the last two years deaths under one week per 1,000 births in Islington were 367, or 21.07 per cent., while for the second week they fell to 103, or nearly 6 per cent. This high death-rate he attributes almost entirely to the ignorance of mothers and the bad conditions in which they live. It is stated that in Islington a great number of men allow their wives to go out to work while they themselves remain idle. Dr. Harris advocates measures to regulate the employment of pregnant women and suckling mothers, and the institution of properly trained health visitors working under the medical officer of health.

Public opinion is being thoroughly roused in Ireland to fight the terrible scourge of tuberculosis, and the determined and organised efforts which are being made to educate the people and to establish measures of prevention and cure augur well for the success of the movement. The Tuberculosis Exhibition, which owes its origin largely to the energy and public spirit of the Countess of Aberdeen, was undoubtely a splendid conception, and the success which has attended the meetings at Dublin and Belfast must have exceeded the expectations of those who were instrumental in its organisation. The popular lectures which have accompanied the exhibition have been attended to overflowing, and the greatest interest has been evinced in the subject by all classes. This alone shows how keenly the people are conscious of the terrible ravages the disease effects in their midst. It is estimated that tuberculosis accounts for more than 12,500 deaths annually in Ireland, and one may reckon that for every death there are at least twenty patients suffering from the disease. In the meantime the medical profession have taken steps to impress upon the authorities the gravity of the matter, and an important deputation, consisting of the presidents of both Colleges and of the Irish Medical Association and many others, waited upon the Lord Lieutenant and Chief Secretary and urged the necessity for measures such as the notification of tuberculosis, the better control of the milk and meat supplies, and provision for sanatoria. The response made to this deputation on behalf of the Government shows that Mr. Birrell is thoroughly in sympathy with the movement. By the co-operation, therefore, of all parties in the country we may hope for some abatement of the evil in the immediate future.

Some important observations have been made by Dr. R. Miller and Dr. W. H. Willcox on gastric conditions in wasted infants. They distinguish three conditions—atrophic dyspepsia, acid dyspepsia, and hypertrophic pyloric stenosis. The latter condition is specially distinguished by the physical signs of gastric peristalsis and a palpable pyloric tumour, but they lay stress on the importance of examining the gastric contents in order to distinguish clearly these three conditions. As a result of their observations on several cases, they find that in atrophic dyspepsia there is a diminished activity of acid and ferment secreting glands, but there is no retention of food and no mucin. In acid dyspepsia there is a marked increase in acidity, but the ferment activity is normal or below normal; there is, however, retention of food, but again no mucin. In hypertrophic stenosis the acidity tends to be below normal, but the ferment activity is increased; there is considerable retention of food and an excessive secretion of mucin. The bearing of these facts upon treatment is obvious. The excess of acid should be met by alkaline gastric lavage and the administration of alkalies. In cases of hypertrophic pyloric stenosis it is pointed out that the excess of mucin causes such firm clotting of the milk that the spasm of the pylorus is thereby increased, and the passage of such clots rendered more difficult. The indication is therefore to give a diet of a non-coagulable nature, such as a whey-and-cream mixture, citrated milk, or milk powders.

Professor Koch's latest report upon the treatment of sleeping sickness with atoxyl is much more encouraging than the previous ones. While the earlier investigations showed that injections of atoxyl caused the disappearance of the trypanosomes in the blood, it was found that they tended to reappear rather rapidly after cessation of the treatment. The attempt to push the treatment by increasing the dose from 0.5 gram to 1 gram at intervals of seven to ten days caused dangerous symptoms, and sometimes permanent blindness, and consequently had to be abandoned. Eventually, however, it has been found that by prolonged treatment over several months with injections of 0.5 gram atoxyl every ten days the disease can be definitely kept in abeyance, and in favourable cases, when treatment is resorted to at an early stage, there seems reasonable hope for effecting a permanent cure. Professor Koch does not find any indication that the trypanosomes acquire a tolerance to the drug as was observed by Ehrlich by experiments on animals, so that the capacity of the parasites to gain a resisting power has not manifested itself as a difficulty to prolonged treatment in the human subject. Professor Koch further points out that, apart from the therapeutic value of the treatment, by keeping the blood of men suffering from the disease free from parasites, they are rendered innocuous as a source of infection to others. Extensive investigations have been carried out to discover some other specific which might prove more efficacious, but hitherto no other drug has been found to possess a therapeutic value at all comparable with atoxyl.

